# Prevalence of HBsAg among reproductive age couples in Chongqing: A population-based, cross-sectional study

**DOI:** 10.1371/journal.pone.0260028

**Published:** 2021-11-15

**Authors:** Qing Chen, Jun Liu, Yang He, Liu Yang, Huiqiang Luo, Yixi Wang, Xuewen Zhang, Ningxiu Li

**Affiliations:** 1 West China School of Public Health and West China Fourth Hospital, Sichuan University, Chengdu, Sichuan Province, China; 2 Chongqing Population and Family Planning Science and Technology Research Institute (NHC Key Laboratory of Birth Defects and Reproductive Health), Chongqing, China; 3 Jining Medical University, Jining, Shandong, China; Middle East Liver Diseases (MELD) Center, Tehran, Iran, ISLAMIC REPUBLIC OF IRAN

## Abstract

Hepatitis B is a leading cause of death worldwide. Here, we performed a large, population-based, cross-sectional study in Chongqing, China from 2011 to 2016 to assess the prevalence of HBsAg among couples of reproductive age, to predict subsequent trends, and to provide evidence for the WHO goal of "the elimination of viral hepatitis as a public health threat by 2030". A total of 386,286 couples aged 20 to 49 years were enrolled in the study. Approximately 14.35% of couples were HBsAg positive, including 95.00% with discordant HBsAg positivity. HBsAg prevalence was higher in men than in women. Among different occupations, the two categories of “houseworker” (female 6.73%, male 9.99%) and “unemployed” (female 6.64%, male 9.94%) showed the highest HBsAg positivity. In different regions, the lowest prevalence appeared in southeastern Chongqing (female 4.87%, male 7.71%). In 2030, the HBsAg positivity rate is expected to be 2.79%, 7.27% and 5.13% in females, males, and the whole population, respectively. According to the trends, this rate would drop to less than 2% in 2034, 2078 and 2051. In conclusion, the HBsAg prevalence in Chongqing is still relatively high compared with that in other parts of western China, especially among reproductive-age men. HBsAg-positive couples should be taken as an important unit of care. Vaccination is necessary before pregnancy if no antibody is found. More attention should be given to people without stable jobs. HBsAg-positive rate will decrease perceptibly by 2030 and will reach the level of low in epidemic areas by 2050.

## Introduction

Hepatitis B virus (HBV), chronic infection is a major risk factor for health because most infections lead to cirrhosis, even hepatocellular carcinoma, which creates heavy burden on society [1]. HBV is a leading cause of death worldwide, with 887,000 related deaths in 2015 [2]. Statistics showed that 257 million persons, or 3.5% of the world’s population, were living with chronic HBV infection in 2015 [2]. China has the world’s largest burden of HBV infection, with 74.6 million people chronically infected [3,4]. The prevalence of HBV infection in couples of reproductive age has been estimated at 10.47% [5]. Assuming that women of reproductive age constitute 25.3% of the world population, adults chronically infected may include 65 million women of childbearing age who can potentially transmit HBV to their babies [6] and other members of their families. China will be a major contributor towards the global elimination of hepatitis B disease by 2030.

Previous studies have examined the seroepidemiology of HBV infection in women [7–11], men [12], or the general population [13–16] in different regions of China, but few studies have focused on couples as a unit. Little is known about the prevalence, incidence, and historical demography of HBV in families. We aimed to carry out a large population-based study in Chongqing to determine the prevalence of HBV in families of reproductive age. Therefore, HBV sero-epidemiological investigation in couples of reproductive age in Chongqing Municipality is necessary to assess the severity of HBV infection in Southwest China and to meet the commitment to reduce new infections by 90% and mortality by 65% (set by the World Health Organization in the “Global Heath Sector Strategy”, the elimination of viral hepatitis as a public health threat by 2030).

## Methods

### Study design and data collection

We conducted a large, population-based, cross-sectional study using a physical check-up program that contains data on couples of reproductive age in Chongqing from 2011 to 2016. Couples who had planned for pregnancies within the next 6 months were enrolled by local community staff. A preconception questionnaire and medical examination were administered by trained local health workers. A unified family health file for the participants was recorded and then entered into a database. The detailed design of this program is described elsewhere [17–19]. Written informed consent was provided by all participants before enrollment. The study was approved by Chongqing Population and Family Planning Science and Technology Research Institute.

### Procedures

Sociodemographic information of the participants was collected via a unified questionnaire, which was excerpted from the National Free Pre-conception Health Examination Project (NFPHEP) family health file. The questionnaire was certified by the ethics review committee of China Maternal and Child Health Research Association. It included age, educational level, occupation, ethnicity, and residential address. Participants from 39 counties were divided into four regions according to their residential addresses and economic status: the central region (including Yuzhong, Dadukou, Jiangbei, Shapingba, Jiulongpo, Nan’an, Beibei, Yubei, Banan), subcentral region (including Fuling, Changshou, Jiangjin, Hechuan, Yongchuan, Nanchuan, Qijiang, Dazu, Tongnan, Tongliang, Rongchang, Bishan, Wansheng), northeastern region (including Wanzhou, Liangping, Chengkou, Fengdu, Dianjiang, Zhongxian, Kaizhou, Yunyang, Fengjie, Wushan, Wuxi) and southeastern region (including Qianjiang, Shizhu, Xiushan, Youyang, Pengshui, Wulong), whose economic status ranged from rich to poor.

A sample of 5 ml venous blood was obtained from each participant at local laboratories affiliated with medical institutions. All sera were separated and tested with quality control mechanisms. Serum specimens were stored at -30° C if they could not be tested immediately. HBV markers were tested using enzyme-linked immunosorbent assay (ELISA) kits. Reagent kits approved by the China Food and Drug Administration were selected by the local laboratories according to their preference. All reagent kits used were tested by the National Center of Clinical Laboratories for Quality Inspection and Detection, with reagents produced by Abbott (Abbott Park, IL, USA) as the reference standard. An external quality assessment (EQA) was performed twice a year to ensure that the sensitivity, specificity, and κ value of the selected reagents were higher than 95% in all the involved counties.

### Definition of outcomes

In our study, positivity of HBsAg indicated that the participant carried HBV and had chronic HBV infection. Wife and/or husband positivity was defined as a positive couple. The positivity of only HBsAb (HBsAb positive while other HBV markers were negative) indicated HBV vaccination-induced immunity.

### Definition of covariates

Participants’ sociodemographic characteristics included age (20–24, 25–29, 30–34, 35–39, 40–44, 45–49), educational level (primary school or less, junior high school, senior high school, college or more), occupation (farmer, worker, service, business, houseworker, teacher/civil servant/staff, unemployed), ethnicity (Han or minorities), and the region of residence (central, subcentral, northeastern and southeastern).

### Statistical analysis

The sociodemographic characteristics of the participants were described as proportions of women, men and couples. The prevalence of HBsAg and its 95% confidence interval (95% CI) for the entire study group, as well as different sociodemographic characteristics, were calculated. Univariate analysis was used to compare the HBsAg difference in every sociodemographic characteristic by the χ^2^ test, and the trend was calculated by the χ^2^ trend test in different age groups and educational groups. Two-sided p < 0.05 was considered statistically significant in all analyses. Multicariate analysis was conducted to compare the differences in age, educational level and ethnic origin of both women and men. The results were used to explain the odds ratio of one variant when other variants were stable. Linear models of y = ax+b were established to estimate the descending rate of HBsAg. R^2^ was calculated to consider the goodness of fit. All analyses were performed with R version 3.6.3 (https://www.r-project.org/).

## Results

### Characteristics of the study population

From Jan 1, 2011, to Dec 31, 2016, we enrolled 388,693 couples in our study with a two-stage stratified cluster sampling method. Among them, 99.6% were aged 20 to 49 years. The serum samples from 386,286 couples (99.8%) were tested, and the data were recorded for the final analysis. Women were aged 27.4±5.73 years, 50.03% had a junior high school education, 47.25% were farmers, and 92.74% were of Han ethnicity. Men were aged 29.7±6.01 years, 49.18% had a junior high school education, 45.61% were farmers, and 92.83% were of Han ethnicity (Table 1).

**Table 1:** Sociodemographic characteristics and prevalence of HBsAg^a^.

	n (%)	Prevalence of HBsAg (%[95%CI])	n (%)	Prevalence of HBsAg (%[95%CI])
	Women		Men	
	386286(100.00%)	6.00%(5.93–6.08)	386286(100.00%)	9.07%(8.98–9.17)
Age (years)				
20–24	137538(35.61%)	5.71%(5.59–5.83)	70824(18.33%)	8.49%(8.28–8.70)
25–29	146282(37.87%)	5.81%(5.69–5.93)	158519(41.04%)	8.74%(8.60–8.88)
30–34	53516(13.85%)	6.33%(6.13–6.54)	77404(20.04%)	9.38%(9.17–9.59)
35–39	28095(7.27%)	7.03%(6.73–7.34)	43833(11.35%)	10.07%(9.79–10.36)
40–44	17228(4.46%)	7.04%(6.66–7.43)	27025(7.00%)	10.07%(9.71–10.43)
45–49	3627(0.94%)	7.31%(6.49–8.21)	8681(2.25%)	9.12%(8.53–9.75)
Occupation				
Farmer	182509(47.25%)	6.10%(5.99–6.21)	176200(45.61%)	9.25%(9.11–9.38)
Worker	35968(9.31%)	6.24%(5.99–6.49)	61115(15.82%)	9.39%(9.16–9.62)
Service	42760(11.07%)	5.52%(5.31–5.74)	37450(9.69%)	8.47%(8.19–8.76)
Business	13655(3.53%)	6.43%(6.03–6.86)	19141(4.96%)	9.69%(9.27–10.12)
Houseworker	20127(5.21%)	6.73%(6.39–7.08)	1752(0.45%)	9.99%(8.64–11.51)
Teachers/civil servants/staff	49992(12.94%)	5.24%(5.05–5.44)	46034(11.92%)	7.74%(7.50–7.99)
Unemployed	30739(7.96%)	6.64%(6.36–6.92)	34148(8.84%)	9.94%(9.63–10.27)
NA’s^b^	10536(2.73%)	5.36%(4.94–5.81)	10446(2.70%)	8.24%(7.73–8.79)
Educational level				
Primary school or less	22958(5.94%)	6.50%(6.19–6.83)	20164(5.22%)	10.22%(9.81–10.65)
Junior high school	193266(50.03%)	6.26%(6.16–6.37)	189976(49.18%)	9.56%(9.42–9.69)
Senior high school	83366(21.58%)	6.01%(5.85–6.17)	87605(22.68%)	8.92%(8.74–9.11)
College or more	86696(22.44%)	5.28%(5.14–5.43)	88541(22.92%)	7.93%(7.75–8.11)
Ethnicity				
Han	358231(92.74%)	6.11%(6.03–6.19)	358590(92.83%)	9.21%(9.12–9.31)
Minorities	28055(7.26%)	4.66%(4.41–4.91)	27696(7.17%)	7.30% (6.99–7.61)

^a^All *p*<0.01;

^b^NA refers to the missing value.

### Seroprevalence of HBsAg

Of the 386 286 couples, 14.35% (14.24%-14.46%) were HBsAg positive. Among these affected couples, 95.00% had a discordant status of HBsAg positivity, including 5.28% (5.21%-5.35%) positive for the wife only and 8.35% (8.26%-8.44%) positive for the husband only. There were 0.73% (0.70%-0.75%) positive results for both the wife and the husband. In total, there were 6.00% (5.93%-6.08%) positive women; and 9.07% (8.98%-9.17%) positive men. HBsAg prevalence was higher in men than in women.

Of the 386,286 women, 31.96% (31.81%- 32.10%) were only HBsAb positive. Among these HBsAb-positive women, 10.77% of their husbands were also infected with HBsAg, which accounted for 37.93% of the 35,053 men infected with HBsAg. Of the 386,286 men, 32.05% (31.90%-32.20%) were only HBsAb positive. Among these HBsAb-positive men, 6.96% of their wives were also infected with HBsAg, which accounted for 37.16% of the 23,191 women infected with HBsAg.

**Fig 1:**
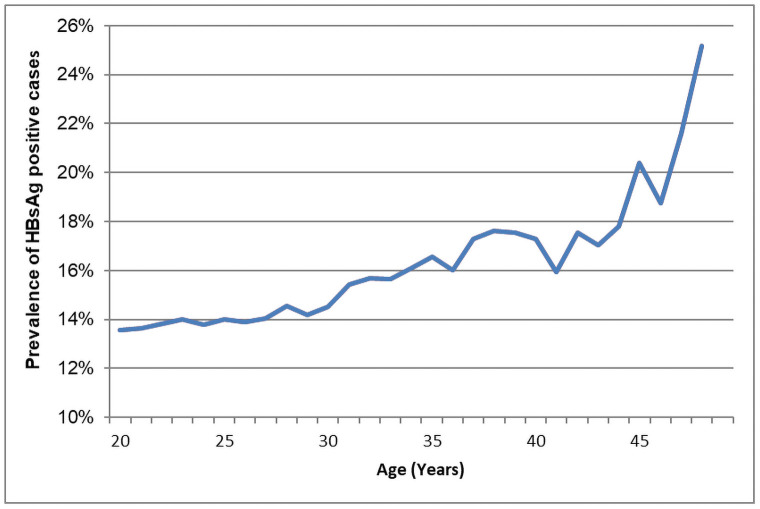
HBsAg prevalence in couples by age.

Women aged 20–24 years had the lowest HBsAg positivity rate (5.71%), and women aged 45–49 years had the highest HBsAg positivity rate (7.31%). The positivity rate increased with age (*χ*^trend2^ = 122.16, p<0.01). Men aged 20–24 years had the lowest HBsAg positivity rate (8.49%), and men aged 35–39 years had the highest HBsAg positivity rate (10.07%). The positivity rate increased with age (*χ*^trend2^ = 112.85, p<0.01). Among the couples, the HBsAg positivity rate also increased with age (Fig 1).

Regarding education, the HBsAg-positive prevalence was the highest among respondents with a primary school education or less (female 6.50%, male 10.22%) and the lowest among those with college degree or more (female 5.28%, male 7.93%). The positivity rate decreased as the education level increased (female *χ*^trend2^ = 104.94, p<0.01; male *χ*^trend2^ = 225.57, p<0.01).

In different occupations, the HBsAg positive prevalence was the lowest in teacher/civil servant/staff (female 5.24%, male 7.74%) and the highest was among those occupied with housework (female 6.73%, male 9.99%), followed by the unemployed (female 6.64%, male 9.94%).

The HBsAg prevalence rate was significantly different in each region (χ^2^ = 357.95, p<0.01). The positivity rate was highest in the subcentral region (female 6.87%, male 10.39%) and lowest in the southeast region (female 4.87%, male 7.71%). In the different ethnic groups, the HBsAg prevalence rate was higher among respondents of Han ethnicity (female 6.11%, male 9.21%) than in the minority group (female 4.66%, male 7.30%, all p<0.01). The proportions of the ethnic groups were different in different regions. In most of the regions, the Han ethnic group was 99% of the population, but in southeastern Chongqing, ethnic minority families accounted for the majority of individuals, and the couple prevalence of HBsAg was significantly lower than the average in Chongqing, especially in couples in which both the husband and the wife were ethnic minorities (Table 2). In logistic regression, as the age of a woman increased by one year, the odds ratio of couple positivity was multiplied by 1.003; as the age of a man increased by one year, the odds ratio of couple positivity was multiplied by 1.015. With the increase in level of education, the odds ratio of couple positivity decreased; the odds ratio of positivity for minorities was lower than for respondents of Han ethnicity.

**Table 2:** Couple prevalence of HBsAg by ethnic origin in southeastern Chongqing.

	N (%)	HBsAg positive couple (n)	Prevalence of HBsAg (%[95%CI])	
FhMh*	10880 (25.75%)	1423	13.08% (12.45–13.73)	χ^2^ = 16.979
FhMm	7335 (17.36%)	898	12.24% (11.51–13.03)	p<0.01
FmMh	5656 (13.39%)	670	11.85% (11.02–12.72)	
FmMm	18383 (43.51%)	2110	11.48% (11.02–11.95)	

*FhMh, female Han and male Han; FhMm, female Han and male minority; FmMh, female minority and male Han; FmMm, female minority and male minority.

### Time trend

**Fig 2:**
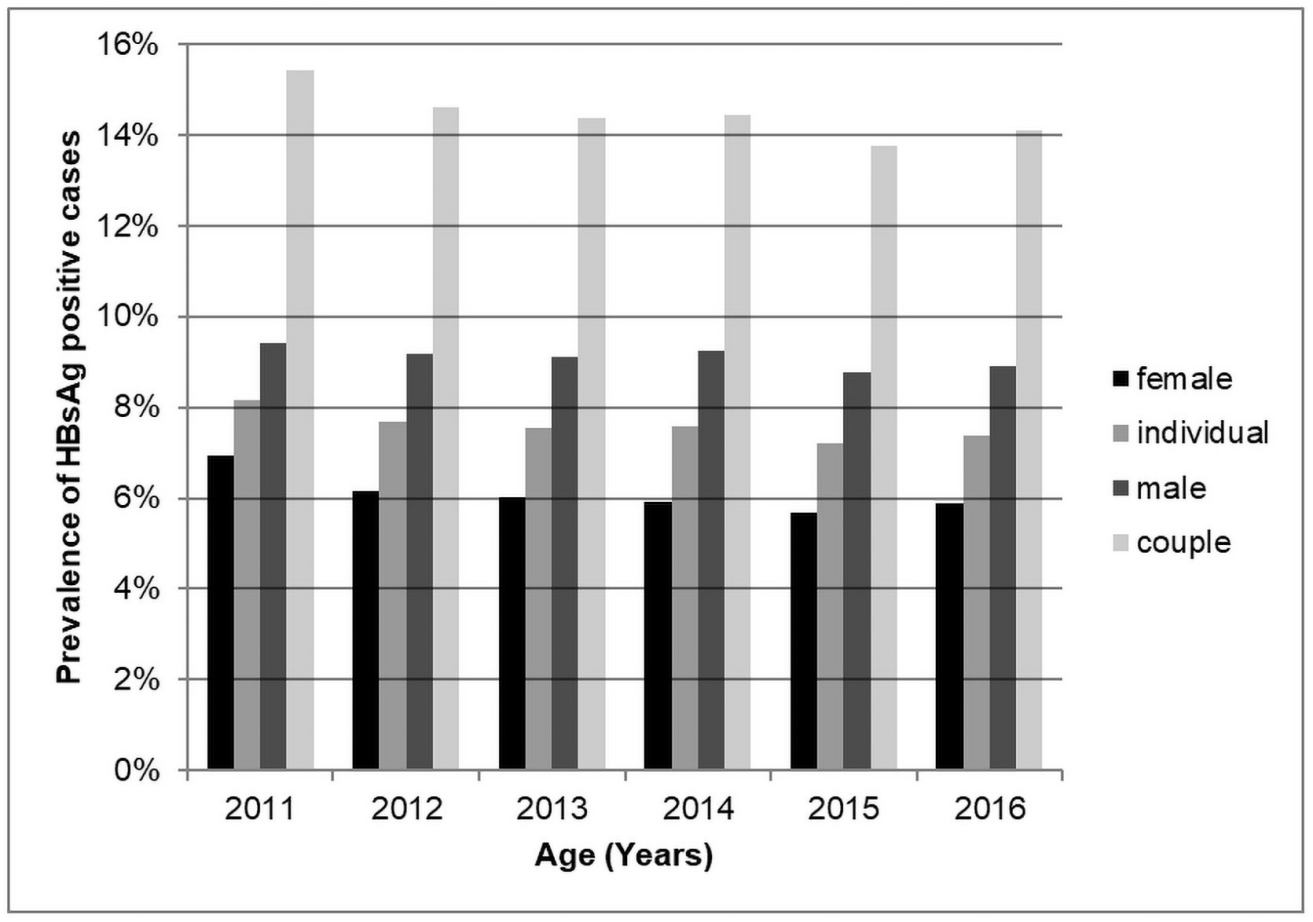
HBsAg prevalence by population and year.

From 2011 to 2016, HBsAg prevalence significantly decreased each year (female *χ*^trend2^ = 30.42, p<0.01; male *χ*^trend2^ = 9.9003, p<0.01, couple *χ*^trend2^ = 29.319, p<0.01, Fig 2). The positive rate of HBsAg was plotted. The scatter showed a linear trend, and the two variables showed a linear regression relationship. Linear models were established from the data.


$$\begin{array}{ll} \text{Female} & \text{y}=-0.0020\text{x}+0.0679,{\text{R}}^{2}=0.6909\\ \text{Male} & \text{y}=-0.0011\text{x}+0.0947,{\text{R}}^{2}=0.7019\\ \text{Individual} & \text{y}=-0.0015\text{x}+0.0813,{\text{R}}^{2}=0.7555 \end{array}$$

From the models, extrapolation showed that the HBsAg positivity rate would be 2.79%, 7.27% and 5.13% in females, males, and the whole population, respectively, by 2030. Then, it would drop to less than 2% in 2034, 2078 and 2051.

## Discussion

Our study demonstrates that 14.35% couples who planned to conceive in Chongqing were affected by HBV infection. Since they were planning to conceive within 6 months, which may increase the risk of transmission through unprotected sex (sex without any contraception measures including condoms that can prevent sexually transmitted disease) between these partners, we considered them (wife and husband together) as a unit. Most of them had a discordant status of HBsAg positivity, which may expose the seronegative partner to a higher risk of infection [20,21], still making it possible to transmit HBV to an unborn child or other members of the family.

Immunization status greatly affects the prevalence of HBV, and the prevalence of HBV also affects vaccination [22]. In our study, among the individuals who had vaccination-induced immunity, the chance of their spouse being HBsAg positive was higher than the average men or women. Among the individuals infected with HBsAg, the proportion of their spouse to have vaccination-induced immunity was also higher than the average men or women. Since this study was a cross-sectional survey, the sequence of infection and vaccination cannot be determined. We assume that some people may have been vaccinated after learning that their spouses have had a confirmed infection, so the results showed that the infection rate of these people was higher than that of the average men and women. However, only one third people have vaccination-induced immunity. Since vaccination is the most effective method of reducing the transmission of HBV [23], we recommend that reproductive-age couples check for hepatitis B antibody before pregnancy. Hepatitis B vaccination is necessary if no antibody to the disease is detected. Family members who have an individual with hepatitis B should be regularly checked for hepatitis B markers. If there is no antibody or the antibody is weak, they should be given the hepatitis B vaccine to prevent the infection.

We found that HBsAg prevalence was higher in men than in women. And the male prevalence was higher than the previous results [12,24]. A recent study in the Lancet showed that the HBsAg prevalence in men in western China was 6.47% [12], but with essentially the same age group in western China, our results revealed a much higher prevalence than that; hence, we infer that the HBsAg prevalence in Chongqing men is at a higher level and higher than in other parts of western China.

The HBsAg prevalence was almost parallel in individuals and in couples with the same sociodemographic characteristics. Our results are similar to those of most other studies. For example, HBsAg prevalence increases with age [14,25,26]; it was higher in the Han ethnic group than in minorities [11,12,27], and it was lower at higher educational levels [11,12,27–29]. As our study revealed, education is a factor affecting HBsAg prevalence, so efforts to educate children, adolescents, and young adults on the transmission of HBV through shared household articles (e.g., toothbrushes, razors, or personal injection equipment) and unsafe sex practices can reduce the transmission of HBV [30–32]. Our study also found that the HBsAg-positive prevalence varies greatly among occupations. The HBsAg-positive prevalence was lower in teachers/civil servants/staff and higher in those occupied with housework or unemployed. The lower number of teachers/civil servants/staff is because they have a higher level of education and higher awareness of the disease [28]. However, the occupation was self-reported by the participants, and those who do not want to let others know about their jobs may report that they stay at home (doing housework or unemployed). Their hidden work suggests that their work may put them at high risk of HBV. On the other hand, those who were HBV positive may have difficulty finding jobs, so more attention should be given to people who do not have stable work.

We found that the proportion of ethnic groups varied greatly in different regions. In most parts of Chongqing, the Han ethnic group accounted for 99% of the population, but in the southeastern region, approximately 60% of residents were minorities. In this region, the positive rate was the lowest in couples who were both members of an ethnic minority while highest in couples who were both of Han ethnicity. This finding indicates that ethnic origin as a minority is a protective factor against HBV in Chongqing.

The World Health Organization calls for "the elimination of viral hepatitis as a public health threat by 2030" [6]. China will be a major contributor towards this goal. And Chongqing is the largest municipal city in China. From 2011 to 2016, the HBsAg prevalence rate significantly decreased every year. The trend analysis also predicted that for women, men and the overall population, the HBsAg positivity rate would decrease every year. We can calculate that the HBsAg positivity rate would drop to a prevalence of less than 2% by 2034, 2078 and 2051 in females, males, and the whole population, respectively. However, we only have data for six years; thus, we cannot speculate over a long timeframe, so the prediction for 2030 and even 2078 may not be accurate. The age range of 20–29 years is the most likely childbearing age range. Men and women in this age group accounted for about 60% and 70% of our survey, respectively. Adolescents will enter childbearing age after a decade, and the older group will pass childbearing age. Taking the effects of infant prophylaxis and early childhood vaccination into account, the prevalence may be lower than we calculated here, and the 2030 goal may be achieved in China ahead of time. The 20–24 years age group in this survey will enter the 35–40 years age group by 2030. At that time, the people in the 20–29 years age group would all be born after 1992, and thus would have received the first hepatitis B vaccine at birth, leading to a much lower HBV infection rate. After the implementation of the “national drug 4 + 7 program”, the price of antiviral drugs has been greatly reduced, which has improved patient access. Thus, more HBsAg-positive patients can be treated. At the same time, the HBsAg positivity rate can be further reduced by effective mother-to-child blocking. The mothers’ participation will greatly reduce the infection rate of hepatitis B virus and accelerate the progress of achieving low epidemic areas.

## Limitations

To our knowledge, this study is the largest of its kind in the Chongqing municipality in southwestern China. However, several limitations exist. First, the target population was only married couples who were planning to conceive within six months, while unmarried people or married people who were not planning to have a baby during the study period were excluded, which may underestimate the prevalence of HBV. Second, the data did not include other risk factors, such as histories of acupuncture treatment or blood transfusions, or risky sexual behavior and sexual orientation that may contribute to transmission. Third, our study design only considered the couples of reproductive age and did not consider their other close family members who may live with them. These data are also extremely important in the study of HBV transmission.

## Conclusions

The HBsAg prevalence in Chongqing is still relatively high compared with that in other parts of western China, especially among reproductive-age men. However, instead of solely focusing on highly infected men, positive couples should be taken as an important unit of care. Vaccination is necessary before pregnancy if no antibody is found. In couples of reproductive age, healthy members in positive families were more likely to have been vaccinated. More attention should be given to people who do not have stable jobs. Living in a minority ethnic region is a protective factor against HBsAg positivity. From 2011 to 2016, the positivity rate of HBsAg decreased year by year. According to the current trend, the positivity rate of HBsAg will decline perceptibly by 2030. It is predicted that the positivity rate of HBsAg will reach low levels in epidemic areas by 2050.

## Supporting information

S1 File Questionnaire (English version). (DOC)

S2 File Questionnaire (Chinese version). (PDF)
