# Supplementary material for: Prevalence of HBsAg among reproductive age couples in Chongqing: A population-based, cross-sectional study
Source: PLoS One. 2021 Nov 15;16(11):e0260028. doi: 10.1371/journal.pone.0260028 (PMC8592447; doi:10.1371/journal.pone.0260028)
Supplement: S1 File — (DOC) [file pone.0260028.s001.doc]

ID：⬜ ⬜ ⬜ ⬜ ⬜ ⬜ ⬜ ⬜ ⬜ ⬜ ⬜ ⬜ ⬜

**National Free Pre-concetion Health Examination Project Family File (excerpt)**

**Sociodemographic Information**

Husband

Ethnic origin Age educational level

occupation ⬜

1 Farmer 2 Worker 3 Service 4 Business 5 Housework 6 Teachers/civil servants/staff 7 Other

County

Wife

Ethnic origin Age educational level

occupation ⬜

1 Farmer 2 Worker 3 Service 4 Business 5 Housework 6 Teachers/civil servants/staff 7 Other

County

**Laboratory test (wife)**

Hepatitis B serological examination 0 negative 1 positive 9 suspicious

⬜HBs-Ag ⬜HBs-Ab ⬜HBe-Ag ⬜HBe-Ab ⬜HBc-Ab

**Laboratory test (husband)**

Hepatitis B serological examination 0 negative 1 positive 9 suspicious

⬜HBs-Ag ⬜HBs-Ab ⬜HBe-Ag ⬜HBe-Ab ⬜HBc-Ab
