# Supplementary material for: Prevalence of HBsAg among reproductive age couples in Chongqing: A population-based, cross-sectional study
Source: PLoS One. 2021 Nov 15;16(11):e0260028. doi: 10.1371/journal.pone.0260028 (PMC8592447; doi:10.1371/journal.pone.0260028)
Supplement: S2 File — (PDF) [file pone.0260028.s002.pdf]

编号：□□□□□□□□□□□□□

国家免费孕前优生健康检查项目技术服务

家 庭 档 案

县级服务机构：_______省________县（市、区）____________________________

乡级服务机构：_______省 _______县（市、区）________乡（镇）____________

基础信息

丈夫姓名________ 民族______ 出生年月______年龄______ 文化程度_________

身份证号码 □□□□□□□□□□□□□□□□□□

职业 □ 1 农民 2 工人 3 服务业 4 经商 5 家务 6 教师/公务员/职员 7 其他____

户口所在地属 _____省 ____市（州）_____县（市、区）____乡（镇）______村（居）

户口性质 □ 1农业户口（含界定为农村居民者） 2非农业户口

妻子姓名________ 民族________ 出生年月_______年龄______文化程度_________

身份证号码 □□□□□□□□□□□□□□□□□□

职业 □ 1 农民 2 工人 3 服务业 4 经商 5 家务 6 教师/公务员/职员 7 其他____

户口所在地属 _____省 ____市（州）_____县（市、区）____乡（镇）______村（居）

户口性质 □ 1农业户口（含界定为农村居民者） 2非农业户口

妻子现住址______省 ______市（州）_____县（市、区）____乡（镇）______村（居）

邮编___________ 结婚时间___________ 联系电话____________________

填写日期__________年_____月_____日 医师签名____________________

国家人口和计划生育委员会编制

国家免费孕前优生健康检查项目专用

孕前检查表（妻子）

临床检验

乙肝血清学检查　0 阴性　1 阳性　9 可疑

□HBs-Ag　□HBs-Ab　□HBe-Ag　□HBe-Ab　□HBc-Ab

检查日期：__________年_____月_____日　　　　医师签名：________________________

孕前检查表（丈夫）

临床检验

乙肝血清学检查　0 阴性　1 阳性　9 可疑

□HBs-Ag　□HBs-Ab　□HBe-Ag　□HBe-Ab　□HBc-Ab

检查日期：__________年_____月_____日　　　　医师签名：________________________

国家免费孕前优生健康检查项目专用
